# The role of the intra-abdominal view in complicated intra-abdominal infections

**DOI:** 10.1186/s13017-019-0232-7

**Published:** 2019-03-29

**Authors:** Matti Tolonen, Ville Sallinen, Ari Leppäniemi, Minna Bäcklund, Panu Mentula

**Affiliations:** 1 Abdominal Center, Department of Abdominal Surgery, Helsinki University Hospital and University of Helsinki, Haartmaninkatu 4, 00290 Helsinki, Finland; 2 Abdominal Center, Department of Transplantation and Liver Surgery, Helsinki University Hospital and University of Helsinki, Helsinki, Finland; 3 Department of Anesthesiology, Intensive Care and Pain Medicine, Division of Intensive Care Medicine, Helsinki University Hospital and University of Helsinki, Helsinki, Finland

**Keywords:** Intra-abdominal infection, Secondary peritonitis, Emergency surgery, Sepsis, Intra-abdominal view, Severe peritonitis

## Abstract

**Background:**

The prognostic role of what a surgeon observes in the abdomen of patients with complicated intra-abdominal infection (cIAI) is largely unknown. The aim of this prospective study was to systemically analyze components of the intra-abdominal view (IAV) and their association to severe complicated intra-abdominal sepsis (SCIAS) or mortality.

**Methods:**

The study cohort consisted of adult patients with cIAI. The operating surgeon filled a paper form describing the intra-abdominal view. Demographics, operative details, and preoperative physiological status were collected. Descriptive, univariate, and multivariate statistical analyses were performed, and a new score was developed based on regression coefficients. The primary outcome was a composite outcome of SCIAS or 30-day mortality, in which SCIAS was defined as organ dysfunctions requiring intensive care unit admission.

**Results:**

A total of 283 patients were analyzed. The primary outcome was encountered in 71 (25%) patients. In the IAV, independent risk factors for the primary outcome were fecal or bile as exudate (odds ratio (OR) 1.98, 95% confidence interval 1.05–3.73), diffuse peritonitis (OR 2.15, 1.02–4.55), diffuse substantial redness of the peritoneum (OR 5.73, 2.12–15.44), and a non-appendiceal source of cIAI (OR 11.20, 4.11–30.54). Based on these factors, an IAV score was developed and its performance analyzed. The area under the receiver operating characteristic for the IAV score was 0.81. The IAV score also correlated significantly with several outcomes and organ dysfunctions.

**Conclusions:**

The extent of peritonitis, diffuse substantial redness of the peritoneum, type of exudate, and source of infection associate independently with SCIAS or mortality. A high IAV score associates with mortality and organ dysfunctions, yet it needs further external validation. Combining components of IAV into comprehensive scoring systems for cIAI patients may provide additional value compared to the current scoring systems.

**Trial registration:**

The study protocol was retrospectively registered on April 4, 2016, right after the first enrolled patient at Clinicaltrials.gov database (NCT02726932).

**Electronic supplementary material:**

The online version of this article (10.1186/s13017-019-0232-7) contains supplementary material, which is available to authorized users.

## Background

Perforation in the gastrointestinal tract results in complicated intra-abdominal infection (cIAI) that may present either as localized peritonitis with or without abscess formation or as diffuse peritonitis [1]. The intra-abdominal view (IAV) in cIAI is traditionally classified into generalized or local and clear, purulent, or fecal according to the appearance of the exudate in the abdomen [2]. Other classifications separate community- or hospital-acquired cIAIs and postoperative or non-postoperative IAIs [3].

In a cIAI, various other findings are often seen in the abdomen, for example, bowel dilatation, bile as exudate, various degrees of fibrin coverage, and redness in the parietal and/or visceral peritoneum.

Even though all surgeons know that a large variety in the IAV exists in patients with cIAI, IAV has not received much attention in previous literature or clinical work. As an example, a patient with acute perforated diverticulitis and a small amount of pus in the pelvis and among small bowel loops, in an otherwise perfectly normally looking abdomen, would be classified as Hinchey III [4]. The same grade of classification would be used in an abdomen full of pus, with dilated bowel, extensive fibrin coverage, and substantially red peritoneal surfaces all over the abdomen. It seems intuitive that recovery paths of these patients would be different and, therefore, all Hinchey III patients cannot be considered equal only based on the existence of purulent peritonitis [5, 6].

There is a wide range of disease severity in patients with cIAI. A term severe complicated intra-abdominal sepsis (SCIAS) to describe sepsis-related organ dysfunctions combined with cIAI specifically due to a disruption in the gastrointestinal tract was introduced in an article by Kirkpatrick et al. [7]. A variety of risk factors for SCIAS or mortality have been identified, preoperative sepsis with associated organ dysfunctions and septic shock being the most important ones [3, 8–10]. Other crucial factors include the physiological reserve of the patient, comorbidities, immunosuppressive medications, and the anatomical derangement caused by the acute disease. There are multiple scoring systems, general and cIAI-specific combining these factors to predict severe outcomes but no one system works satisfyingly [11]. These systems include limited data of the IAV.

The aim of this prospective study was to systematically analyze components of IAV and their association to SCIAS or 30-day mortality.

## Methods

### Patients and setting

This study was conducted as a prospective cohort study in a single academic center that serves both as a secondary and a tertiary referral center. Institutional ethics committee and review board approved the study design. A written informed consent was obtained from all the patients included in the study. The study period was 2 years, starting on 31 March 2016 and ending on 31 March 2018.

Eligible patients were adult (over 18 years old) patients undergoing surgical intervention because of a cIAI. Patients with pancreatitis, acute mesenteric ischemia, or trauma were excluded. During the study period, electronic operating room logs were manually browsed for all abdominal emergency operations to verify that all patients with a cIAI were identified.

### Intra-abdominal view

Data regarding intra-abdominal findings was collected on a paper sheet form filled by the operating surgeon after the operation (Additional file 1). In the form, the abdomen was divided into six areas: right and left upper supramesocolic area, right and left lower and paracolic area, mid-abdomen small bowel area, and pelvic area (below the promontorium). Small and/or large bowel dilatation was recorded as well as the type of exudate (clear, purulent, fecal, bile) by area. If any area contained fecal or bile exudate, the peritonitis was classified as fecal or bile peritonitis, respectively. If both were present, the peritonitis was classified according to the predominant exudate. In addition, the amount of fibrin by area (none, mild, or substantial) and redness of the peritoneum by area (none, mild, or substantial) were recorded. Further, the localization of infection in the peritoneum by area (parietal and/or visceral, excluding the pelvic area) was recorded. The difference between mild and substantial finding was a subjective estimation based on clinical judgment and experience.

### Definitions

Comorbidities were classified according to the Charlson Comorbidity Index [12]. The classification of sepsis was recorded as in the Sepsis-III guidelines, i.e., sepsis is an acute change of total Sequential Organ Failure Assessment (SOFA) score two or more and septic shock is sepsis with persisting hypotension requiring vasopressors to maintain mean arterial pressure ≥ 65 mmHg and a persistent serum lactate level > 2 mmol/l despite adequate volume resuscitation [10]. Other reported scores were the Mannheim Peritonitis Index, World Society of Emergency Surgery (WSES) Sepsis Severity Score for patients with cIAIs, Acute Physiology And Chronic Health Evaluation II (APACHE-II), Clavien-Dindo complication classification, and the SOFA scores in patients admitted to the ICU [2, 13–16]. For the Clavien-Dindo classification, the most serious in-hospital complication was used. Preoperative organ dysfunctions (grade IV) or antibiotic treatment for cIAI were not documented as complications. Only new onset organ dysfunctions or complications that significantly contributed to the worsening of preoperative organ dysfunctions were classified as grade IV [17]. Immunosuppression was defined according to the WSES Sepsis Severity Score, i.e., chronic use of glucocorticoids, immunosuppressive medication, chemotherapy within 30 days, or lymphatic disease. The primary outcome was a composite outcome of SCIAS or 30-day mortality. SCIAS translated to ICU admission due to organ dysfunctions. Similar definition has also been used in a recent study [11].

### Statistical analyses

Descriptive statistics are demonstrated in number, percentage, mean, median, and interquartile range (IQR), where appropriate. Univariate analyses were made using binary logistic regression for categorical variables, Mantel-Haenszel linear-by-linear association chi-squared test for ordinal variables, and Mann-Whitney *U* test or Kruskal-Wallis test for continuous variables without normal distribution, where appropriate. Nonparametric correlations between continuous variables were tested with Spearman’s rho test. Multivariate analysis was conducted using binary logistic regression. Multivariate goodness-of-fit was tested using Hosmer-Lemeshow test, and model performance was tested using Nagelkerke *R*^2^. Variables for multivariate analysis were chosen from the results of the univariate analysis. After logical reclassification in some variables, the most significant variable from each IAV component (perforated organ, extent of peritonitis, bowel dilatation, type of exudate, fibrin coverage, and redness in peritoneum) was chosen. Different logistic regression models were tested with different combinations of variables, and the model with the highest Nagelkerke *R*^2^ value was chosen. The scoring system was built according to the regression coefficients in the logistic regression equation. A receiver operating characteristic (ROC) curve was plotted, and area under ROC curve (AUROC) calculated. Two-tailed *P* value below 0.05 was considered significant, and odds ratio (OR) values are presented with 95% confidence intervals (CI). Analyses mentioned above were performed using a SPSS© Statistics version 22 for Mac (IBM©, Armonk, NY, USA). Internal validation on logistic model was done with bootstrapping and by calculating optimism-adjusted AUC [18]. This analysis was done using R (R Foundation for Statistical Computing, Vienna, Austria. URL https://www.R-project.org/.) with pROC package [19] and R function for optimism-adjusted AUC [20].

### Power calculation

Power calculations for this study were based on the primary outcome measure, i.e., SCIAS or 30-day mortality. In order to perform a statistically appropriate multivariate analysis with seven variables, we calculated the need for a minimum of 49 patients with the primary outcome event. Based on previous reports, 10–25% of patients with cIAI will meet the primary outcome [8, 13]. For power calculation purposes, we estimated that 17% of cIAI patients would meet the primary outcome event resulting in a need to recruit at least 288 patients.

## Results

**Fig. 1:**
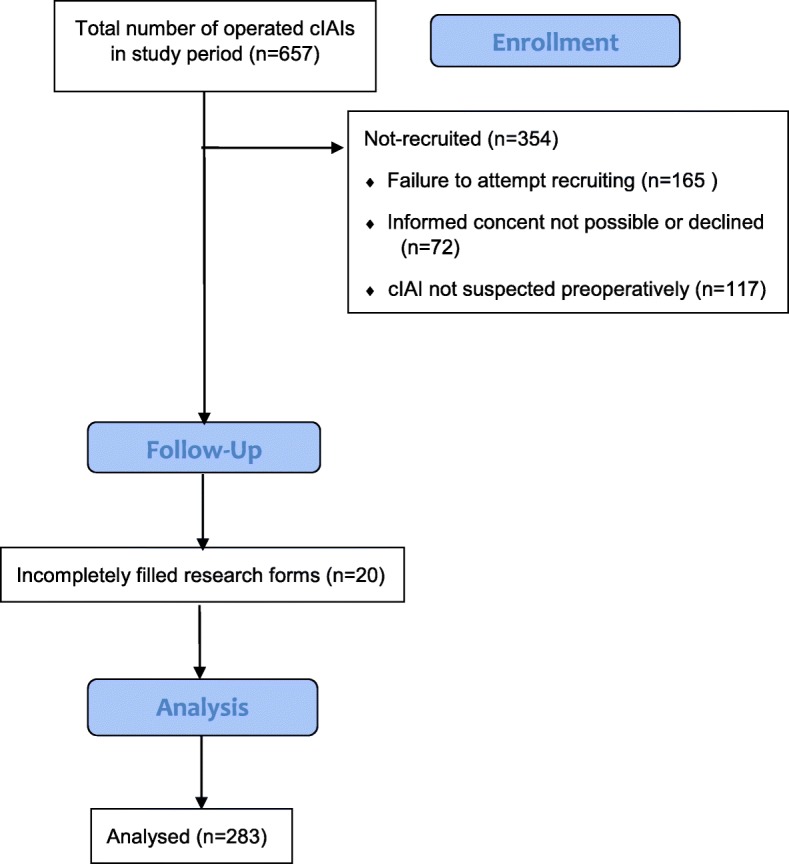
Patient flow chart

During the 2-year study period, there were 657 operated patients with cIAIs filling the inclusion; please see the modified flow diagram (Fig. 1). The most common reasons for not recruiting patients were failure to attempt recruiting and not suspecting cIAI preoperatively.

A total of 283 patients with properly filled IAV form were included and analyzed. Patient characteristics, together with the intra- and postoperative data, are presented in Table 1, describing the study cohort. Briefly, 132 (47%) were male with a median age of 64 years. Sepsis was present in 60 (21%) and septic shock in 19 (7%) patients preoperatively. The most common sources of cIAI were the appendix (*n* = 109, 39%) and colorectal (*n* = 101, 36%). The median length of hospital stay was 6 days, and 57 (20%) patients were admitted to the ICU with a median peak SOFA score of 8. All patients admitted to the ICU had a SOFA score of at least 1. Twenty-nine (10%) patients died within 30 days, and SCIAS or mortality was encountered in 71 (25%) patients.

**Table 1:** Patient characteristics

Preoperative	*n* (%) (total *n* = 283)
Sex, male	132 (47)
Age, years	64 (49–74)^a^
BMI	21 (19–24)^a^
Charlson Comorbidity Index	1 (0–5)^a^
Immunosuppression	54 (19)
Malignant diseases	
Local solid malignant tumor	35 (12)
Solid metastatic tumor or lymphoma	45 (16)
Sepsis classification	
No sepsis	204 (72)
Sepsis	60 (21)
Septic shock	19 (7)
Hospital-acquired cIAI	62 (22)
Intraoperative	*n* (%)
Delay from symptoms to operation < 24 h	79 (28)
Perforated organ	
Gastroduodenal	32 (11)
Small bowel	30 (11)
Colorectal	101 (36)
Appendix	109 (39)
Gallbladder	11 (4)
Operation	
Laparotomy	152 (54)
Laparoscopy	110 (39)
Converted from laparoscopy to laparotomy	21 (7)
Open abdomen	5 (2)
Postoperative peritonitis	38 (13)
Mannheim Peritonitis Index	26 (22–32)^a^
WSES Sepsis Severity Score	6 (3–9)^a^
APACHE-II	9 (6–15)^a^
Postoperative	*n* (%)
CRP, highest value, mg/l	304 (244–364)^a^
Reoperations	36 (13)
Postoperative abscess	28 (10)
Clavien-Dindo classification, in-hospital	
0	111 (39)
1	47 (17)
2	38 (13)
3a	28 (10)
3b	25 (9)
4a	7 (2)
4b	3 (1)
5	24 (8)
Length of hospital stay, days	6 (3–11)^a^
ICU admission	57 (20)
Renal replacement therapy	2 (1)
Peak SOFA score at ICU	8 (6–10)^a^
Prolonged (> 12 h) recovery room stay	25 (9)
Mortality, 30 days	29 (10)
SCIAS or 30-day mortality	71 (25)
Mortality, 90 days	37 (13)

*Abbreviations*: *BMI* body mass index, *cIAI* complicated intra-abdominal infection, *WSES* World Society of Emergency Surgery, *APACHE* Acute Physiology and Chronic Health Evaluation, *CRP* C-reactive protein, *ICU* intensive care unit, *SCIAS* severe complicated intra-abdominal sepsis

^a^Continuous variables are presented as median (interquartile range)

Several different components of the IAV were associated with the primary outcome (Table 2). Some components of the IAV were reclassified according to the results to build the multivariate analysis (marked with “*” in Table 2). Non-appendiceal source of cIAI, diffuse peritonitis (four or more out of six areas), dilatation of the colon only, fecal or bile as exudate, substantial fibrin deposits found in five or more areas, and substantial redness found in four or more areas were chosen for the multivariate analysis. Correlations between continuous variables were tested, and all results were significant (*P* < 0.001). Correlation coefficients for the amount of substantial redness by area were 0.433 for the amount of substantial fibrin by area and 0.385 for the extent of peritonitis by area. For the amount of substantial fibrin by area and extent of peritonitis by area, the correlation coefficient was 0.293.

**Table 2:** Univariate binary logistic regression of components of the intra-abdominal view for severe complicated intra-abdominal sepsis (SCIAS) or 30-day mortality

Risk factor	*n* (%)	SCIAS or 30-day mortality, *n* (%)	OR (95% CI)	*P* value	*B*
Perforated organ					
Appendix	109 (39)	5 (5)	Reference	*< 0.001*	
Gallbladder	11 (4)	4 (36)	11.89 (2.60–54.42)	*0.001*	2.475
Colorectal	101 (36)	37 (37)	12.03 (4.49–32.18)	*< 0.001*	2.487
Gastroduodenal	32 (11)	12 (38)	12.48 (3.96–39.33)	*< 0.001*	2.524
Small bowel	30 (11)	13 (43)	15.91 (5.03–50.33)	*< 0.001*	2.767
Non-appendiceal source*	174 (62)	66 (38)	12.71 (4.93–32.81)	*< 0.001*	2.542
Extent of peritonitis by number of affected areas					
1	30 (11)	4 (13)	Reference	*0.001*	
2	47 (17)	7 (15)	1.14 (0.30–4.28)	0.849	0.129
3	29 (10)	1 (3)	0.23 (0.02–2.21)	0.204	− 1.460
4	32 (11)	8 (25)	2.17 (.58–8.13)	0.252	0.773
5	32 (11)	7 (22)	1.82 (0.47–6.99)	0.383	0.599
6	113 (40)	44 (39)	4.15 (1.35–12.69)	*0.013*	1.422
Diffuse peritonitis (≥ 4 areas)*	177 (63)	59 (33)	3.92 (1.99–7.71)	*< 0.001*	1.365
Bowel dilatation					
No bowel dilatation	164 (58)	35 (21)	Reference	0.097	
Small bowel only	77 (27)	19 (25)	1.21 (0.64–2.29)	0.563	0.188
Colon only*	19 (7)	8 (42)	2.68 (1.00–7.17)	*0.050*	0.986
Small bowel and colon	23 (8)	9 (39)	2.37 (0.95–5.93)	0.065	0.863
Type of exudate					
Clear	16 (6)	3 (19)	Reference	*0.001*	
Purulent	160 (57)	27 (17)	0.88 (0.24–3.30)	0.849	− 0.128
Fecal	85 (30)	30 (35)	2.36 (0.62–8.95)	0.206	0.860
Bile	22 (8)	11 (52)	4.33 (0.96–19.58)	0.057	1.466
Fecal or bile*	107 (38)	41 (38)	3.02 (1.74–5.26)	*< 0.001*	1.106
Extent of fibrin by number of affected areas					
0	37 (13)	15 (41)	Reference	*< 0.001*	
1	77 (27)	12 (16)	0.27 (0.11–0.67)	*0.004*	− 1.306
2	46 (16)	7 (15)	0.26 (0.09–0.74)	0.120	− 1.335
3	28 (10)	3 (11)	0.18 (0.05–0.69)	0.130	− 1.737
4	31 (11)	6 (19)	0.35 (0.12–1.06)	0.640	− 1.044
5	24 (9)	10 (42)	1.05 (0.37–2.98)	0.930	0.047
6	39 (14)	18 (46)	1.26 (0.51–3.12)	0.622	0.229
Amount of substantial fibrin by number of affected areas					
0	140 (49)	33 (24)	Reference	*0.016*	
1	72 (25)	12 (17)	0.65 (0.31–1.35)	0.246	− 0.433
2	25 (9)	6 (24)	1.02 (0.38–2.78)	0.963	0.024
3	19 (7)	7 (37)	1.89 (0.69–5.20)	0.216	0.637
4	10 (4)	2 (20)	0.81 (0.16–4.01)	0.797	− 0.210
5	6 (2)	4 (67)	6.49 (1.14–37.01)	*0.035*	1.869
6	11 (4)	7 (64)	5.67 (1.56–20.59)	*0.008*	1.736
Substantial fibrin ≥ 5 or more areas*	17 (6)	11 (65)	6.29 (2.2–17.73)	*< 0.001*	1.840
Extent of redness by number of affected areas					
0	17 (6)	8 (47)	Reference	*< 0.001*	
1	41 (14)	6 (15)	0.19 (0.05–0.70)	*0.012*	− 1.646
2	55 (19)	7 (13)	0.16 (0.05–0.57)	*0.004*	− 1.808
3	32 (11)	3 (9)	0.12 (0.03–0.53)	*0.006*	− 2.151
4	32 (11)	7 (22)	0.32 (0.09–1.12)	0.074	− 1.155
5	27 (10)	7 (26)	0.39 (0.11–1.42)	0.155	− 0.932
6	78 (28)	33 (42)	0.83 (0.29–2.37)	0.720	− 1.192
Amount of substantial redness by number of affected areas					
0	125 (44)	26 (21)	Reference	*< 0.001*	
1	73 (26)	10 (14)	0.60 (0.27–1.34)	0.214	− 0.504
2	34 (12)	10 (29)	1.59 (0.68–3.73)	0.290	0.462
3	21 (7)	5 (24)	1.19 (0.40–3.55)	0.755	0.174
4	9 (3)	5 (56)	4.76 (1.19–18.99)	*0.027*	1.560
5	3 (1)	2 (67)	7.62 (0.66–87.29)	0.103	2.030
6	18 (6)	13 (72)	9.90 (3.24–30.29)	*< 0.001*	2.293
Substantial redness ≥ 4 or more areas*	30 (11)	20 (67)	7.92 (3.49–17.97)	*< 0.001*	2.070
Localization of cIAI non-parietal (only visceral)	49 (17)	11 (22)	0.84 (0.40–1.75)	0.639	− 1.094

Method: enter, all variables categorical. Italics indicate statistical significance

*Abbreviations*: *OR* odds ratio, *B* regression coefficient, *cIAI* complicated intra-abdominal infection

*Included in multivariate analysis

In the multivariate binary logistic regression analysis, four components of the IAV were found independently significantly associated with the primary outcome (Table 3). In an ascending OR order, these factors were fecal or bile as exudate, diffuse peritonitis, substantial redness in four or more areas, and a non-appendiceal source. AUROC for this logistic model was 0.812 (95% CI 0.761–0.865). Internal validity of the logistic model was tested with bootstrapping. Distribution of the AUROC value in the bootstrap sample is shown in Additional file 2. Optimism-adjusted AUROC for the logistic model was 0.802. Hosmer-Lemeshow goodness-of-fit test significance was 0.36 showing the model had adequate fit. Model performance was tested using Nagelkerke *R*^2^ with a result of 0.36.

**Table 3:** Multivariate binary logistic regression of the intra-abdominal view for severe complicated intra-abdominal sepsis (SCIAS) or 30-day mortality

Risk factor	SCIAS or 30-day mortality OR (95% CI)	*P* value	*B*	IAV score
Exudate fecal or bile	1.98 (1.05–3.73)	0.034	0.685	1
Diffuse peritonitis (≥ 4 areas)	2.15 (1.02–4.55)	0.045	0.767	1
Substantial redness (≥ 4 areas)	5.73 (2.12–15.44)	0.001	1.745	2
Non-appendiceal source	11.20 (4.11–30.54)	< 0.001	2.416	3

Method: forward LR, all variables categorical

*Abbreviations*: *OR* odds ratio, *CI* confidence interval, *B* regression coefficient, *IAV* intra-abdominal view (score = 1.3 × *B* to nearest integer)

**Fig. 2:**
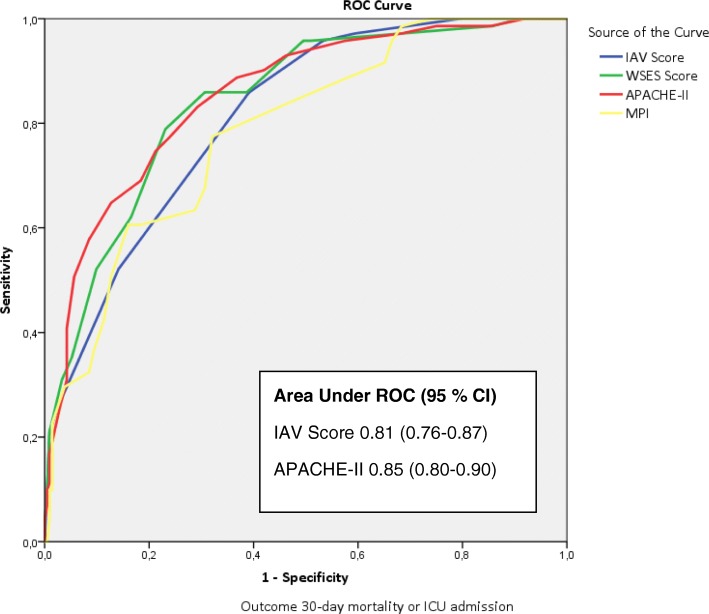
Receiver operating characteristic (ROC) curve for the intra-abdominal view (IAV) score and comparisons to other scores. Abbreviations: WSES World Society of Emergency Surgery, APACHE Acute Physiology And Chronic Health Evaluation, MPI Mannheim Peritonitis index, CI confidence interval

The IAV score is also presented in Table 3. Regression coefficients were multiplied by 1.3, and the nearest integer was the score for each variable. AUROC for IAV score predicting SCIAS or 30-day mortality was 0.81, and it remained unchanged from the original logistic model. As shown in Fig. 2, the IAV score performed nearly as good as other scores in predicting SCIAS or mortality. With the help of the ROC curve (Fig. 2), two cutoff points were chosen to divide patients to low (0–2 points), medium (3–5 points), and high score (6–7 points) groups. These groups were compared for various different outcomes (Table 4).

**Table 4:** The intra-abdominal view (IAV) score evaluation; low, middle, and high scores with various outcomes

	All patients	Low score	Medium score	High score	*P* value*
IAV score	4 (1–4)**	0–2	3–5	6–7	
Number of patients, *n* (%)	283 (100)	102 (36)	158 (56)	23 (8)	
Length of stay, days**	6 (3–10)	3 (2–5)	8 (6–13)	10 (6–18)	< 0.001
Clavien-Dindo ≥ 3, *n* (%)	87 (31)	14 (14)	62 (39)	11 (48)	< 0.001
ICU admission, *n* (%)	57 (20)	2 (2)	39 (25)	16 (70)	< 0.001
Mortality, 30 days, *n* (%)	29 (10)	1 (1)	22 (14)	6 (26)	< 0.001
SCIAS or 30-day mortality, *n* (%)	71 (25)	3 (3)	50 (32)	17 (74)	< 0.001

*Abbreviation*: *ICU* intensive care unit

*P values are calculated using linear-by-linear association for dichotomous variables and Kruskal-Wallis test for continuous variable

**Continuous variables are presented as median (interquartile range)

**Fig. 3:**
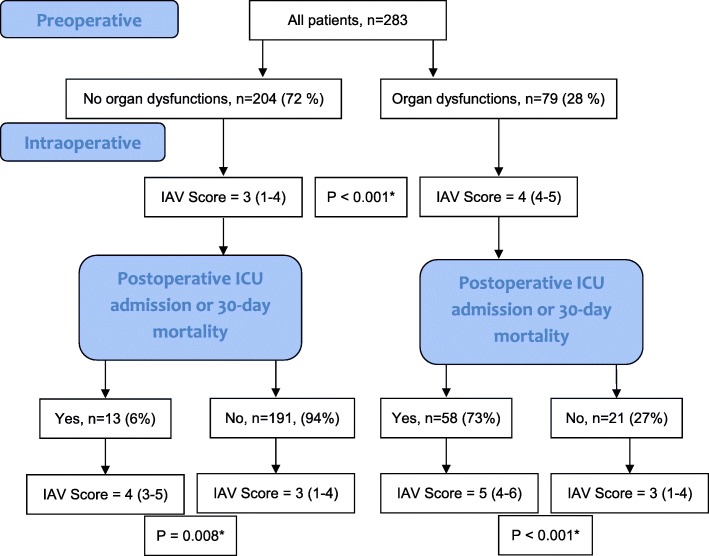
The intra-abdominal view (IAV) score correlations with pre- and postoperative organ dysfunctions. Legend: IAV scores are presented as median (interquartile range), *Mann-Whitney *U* test

Because of the dynamic nature of organ dysfunctions, an additional analysis was made to evaluate whether the IAV score could predict the course of organ dysfunction (Fig. 3). Patients with preoperative organ dysfunctions had a higher median IAV score (4, IQR 4–5) compared to patients without organ dysfunctions (3, IQR 1–4), *P* < 0.001. Of the 204 patients without preoperative organ dysfunctions, 13 (6%) were admitted to the ICU or died, and these patients had a significantly higher IAV score (4, IQR 3–5) compared to the patients who recovered without ICU treatment (3, IQR 1–4), *P* = 0.008. On the other hand, patients whose preoperative organ dysfunctions resolved quickly without ICU treatment had a lower IAV score (3, IQR 1–4) compared to the ones who were admitted to the ICU or died (5, IQR 4–6), *P* < 0.001.

### Subgroup analysis

Due to the much better prognosis in acute appendicitis as the source of cIAI, we performed a subgroup analysis with a non-appendiceal source of cIAI. There were 174 patients, of which 27 (16%) died within 30 days, 53 (30%) were admitted to the ICU, and 66 (38%) had a SCIAS. A multivariate analysis was performed with the Table 3 variables, without source of infection, using the enter method. The results were substantial redness in ≥ 4 areas (OR 5.09, 95% CI 1.71–15.13, *P* 0.003, *B* 1.628), fecal or bile as exudate (OR 2.06, 95% CI 1.06–4.03, *P* 0.034, *B* 0.725), and diffuse peritonitis (OR 2.14, 95% CI 0.97–4.72, *P* 0.059, *B* 0.761). Hosmer-Lemeshow test showed a non-significant value of 0.91 and Nagelkerke *R*^2^ 0.19.

## Discussion

This pioneer prospective study of the IAV in patients with cIAI recognized several independent factors associated with SCIAS or 30-day mortality. Based on these factors, an IAV score was developed and its performance further analyzed.

We chose a composite outcome of either ICU admission due to acute organ dysfunctions (=SCIAS) or 30-day mortality as the primary outcome. All patients admitted to the ICU had acute organ dysfunctions. With this outcome, all patients with a severe outcome were recognized, since some patients may die without being admitted to the ICU. This may be due to ICU admission refusals, or a more surprising death without diagnosed previous organ dysfunctions.

The results from the multivariate analysis are somewhat expected. A completely new finding was that having substantial redness in four or more out of six areas in the abdomen was independently associated with the primary outcome. Intuitively, it seems logical. The inflammatory response of the peritoneum is characterized by enhanced vascular perfusion, accumulation of macrophages with subsequent attraction of more immune cells, and release of pro- and anti-inflammatory mediators [21–23]. The stronger the inflammation process, the more visible the redness of the peritoneum. Patients with appendicitis have substantially better prognosis compared to other cIAIs [3, 13]. Even when adjusting for other IAV factors, patients having other sources than appendicitis was the risk factor with the highest OR. In future cIAI studies, it should be considered if appendicitis should be studied as a separate entirety with different outcome measures due to a much less severe course of the disease. Having a diffuse, rather than localized cIAI, the risk for SCIAS or 30-day mortality increased threefold. This risk seems obvious and has also been identified by the previously existing scoring systems [2, 13]. When the type of exudate was fecal or bile, compared to clear or purulent, there was a more than twofold increase for the primary outcome. This factor is taken into account in the Mannheim Peritonitis Index [2] but not in other cIAI-specific or sepsis scoring systems [11, 13].

Fibrin coverage was not independently associated with the primary outcome. Fibrin correlated with redness but the redness was more significant in multivariate analysis and therefore fibrin did not stand out as an independent risk factor. Venous blood flow from the visceral peritoneum goes first through the portal vein into the liver, whereas parietal venous flow goes directly to systemic circulation. Also, the visceral and parietal peritoneum have distinct innervations and the surface area of the visceral peritoneum is much larger than that of the parietal peritoneum [24]. However, localization of the cIAI only in the visceral peritoneum did not correlate with outcome.

The values of Nagelkerke *R*^2^ 0.36 and AUROC 0.81 for the IAV score clearly show that IAV only accounts for a limited, although substantial, part of prognostic factors for SCIAS or 30-day mortality. However, it is quite surprising that the AUROC for the IAV score is of the same magnitude as in the more comprehensive scoring systems, since organ dysfunctions and comorbidities are not included in the model [11]. These results emphasize the role of the surgeons’ perception of cIAI disease severity when looking into the abdomen while operating. A subgroup analysis of patients with a non-appendiceal source of cIAI showed very similar results and was not considered to provide any additional value.

The IAV score was tested by dividing patients into three score groups (Table 4), and it was found to correlate significantly with a variety of different outcomes. In Fig. 3, it is shown that patients who improve quickly, without ICU treatment, from preoperative organ dysfunctions have a lower IAV score. In addition, patients without preoperative organ dysfunctions who eventually develop SCIAS or die have a higher score than patients who recover without organ dysfunctions. It is not surprising that the worse the macroscopic view of the peritonitis is, the worse the outcome. Nevertheless, this is the first study that shows and quantifies it.

Interestingly, in the univariate analyses, when the extent of fibrin and the redness by area was zero, the risk for the primary outcome was high, resulting in a U-shaped curve within the variable. The reason for this finding can only be speculated and it warrants further research, but the phenomenon might be associated with an impaired immune response to cIAI.

It is well known that organ dysfunctions are the most important risk factors for poor outcome [8, 11, 13]. Another major factor not included in this study’s model is the comorbidities. These factors were not included in this study since the focus of this study was an in-depth analysis of the IAV. In future studies, including components of the IAV to a more comprehensive scoring system could provide a better scoring system than the current ones.

This study has some limitations. This was a prospective single-center study with a limited number of patients. Less than half of the recruitable patients during the study period were included in the study. The evaluation of the amount of fibrin deposits as well as redness of the peritoneum was based on subjective evaluation leading most likely into some interobserver variability. Also, the IAV score has not been externally validated.

## Conclusions

Classification of peritonitis should not be based only on the type of the exudate or presence of diffuse peritonitis. An IAV score, which includes fecal or bile as exudate, diffuse peritonitis, substantial redness in four or more of the six areas of the abdomen, and non-appendiceal source of cIAI, may provide a simple method to classify patients with cIAI. The IAV score predicts various outcomes well and correlates with preoperative organ dysfunctions and the development of postoperative organ dysfunctions. The concept of including more variables of the IAV to cIAI scoring systems might provide additional value in assessing individual patient disease severity and outcome. However, the IAV score needs external validation before further implementations.

## Additional files

Additional file 1: Paper sheet for the collection of the intra-abdominal findings (DOCX 468 kb)

Additional file 2: Bootstrapping. Legend: Distribution of AUC value in the bootstrap sample (auc.boot) and the distribution of the AUC value deriving from the model fitted to the bootstrap samples and evaluated on the original sample (auc.orig). The blue line represents apparent AUC and the red line AUC adjusted for optimism. (DOCX 65 kb)

## Abbreviations

APACHE-II: Acute Physiology And Chronic Health Evaluation II
AUROC: Area under the receiver operating characteristic curve
CI: Confidence interval
cIAI: Complicated intra-abdominal infection
IAV: Intra-abdominal view
ICU: Intensive care unit
IQR: Interquartile range
OR: Odds ratio
ROC: Receiver operating characteristic
SCIAS: Severe complicated intra-abdominal sepsis
SOFA: Sequential Organ Failure Assessment
WSES: World Society of Emergency Surgery
