# Supplementary material for: The role of the intra-abdominal view in complicated intra-abdominal infections
Source: World J Emerg Surg. 2019 Mar 29;14:15. doi: 10.1186/s13017-019-0232-7 (PMC6441193; doi:10.1186/s13017-019-0232-7)
Supplement: Supplementary file 1 — Paper sheet for the collection of the intra-abdominal findings (DOCX 468 kb) [file 13017_2019_232_MOESM1_ESM.docx]

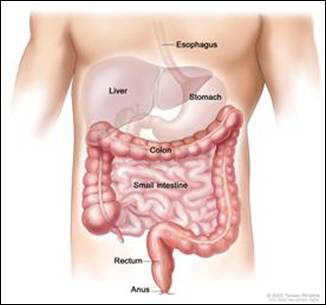


Date of operation

Patient name and ID

**Bowel dilatation?**

No Small bowel Colon

**Pelvic area**

**Exudate?**  No Clear Purulent

Fecal Bile

**Fibrin?**

No Mild Substantial

**Redness of peritoneum?** No Mild Substantial

**Left upper abdomen**

**Exudate?**  No Clear Purulent

Fecal Bile

**Fibrin?**

No Mild Substantial

**Localization of infection in peritoneum?**

Parietal Visceral Both

**Redness of peritoneum?** No Mild Substantial

**Left lower abdomen**

**Exudate?**  No Clear Purulent

Fecal Bile

**Fibrin?**

No Mild Substantial

**Localization of infection in peritoneum?**

Parietal Visceral Both

**Redness of peritoneum?** No Mild Substantial

**Right upper abdomen**

**Exudate?**  No Clear Purulent

Fecal Bile

**Fibrin?**

No Mild Substantial

**Localization of infection in peritoneum?**

Parietal Visceral Both

**Redness of peritoneum?** No Mild Substantial

**Mid-abdomen, small bowel**

**Exudate?**  No Clear Purulent

Fecal Bile

**Fibrin?**

No Mild Substantial

**Localization of infection in peritoneum?**

Parietal Visceral Both

**Redness of peritoneum?** No Mild Substantial

**Right lower abdomen**

**Exudate?**  No Clear Purulent

Fecal Bile

**Fibrin?**

No Mild Substantial

**Localization of infection in peritoneum?**

Parietal Visceral Both

**Redness of peritoneum?** No Mild Substantial
