# Supplementary figures and images for: The role of the intra-abdominal view in complicated intra-abdominal infections
Source: World J Emerg Surg. 2019 Mar 29;14:15. doi: 10.1186/s13017-019-0232-7 (PMC6441193; doi:10.1186/s13017-019-0232-7)

Additional file 2.


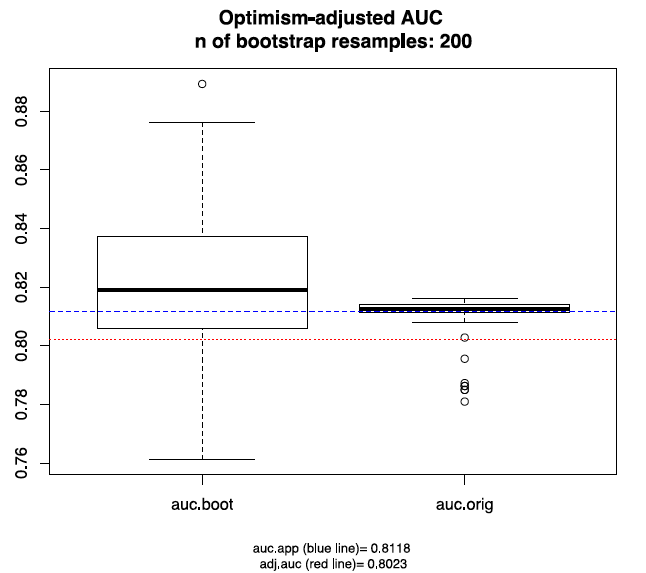

Supplement: Supplementary file 2 — Bootstrapping. Legend: Distribution of AUC value in the bootstrap sample (auc.boot) and the distribution of the AUC value deriving from the model fitted to the bootstrap samples and evaluated on the original sample (auc.orig). The blue line represents apparent AUC and the red line AUC adjusted for optimism. (DOCX 65 kb) [file 13017_2019_232_MOESM2_ESM.docx]
